# Interaktive intraoperative Annotation chirurgischer Anatomie in der studentischen Ausbildung zur Unterstützung der Lerneffizienz und -motivation

**DOI:** 10.1007/s00106-022-01187-5

**Published:** 2022-06-04

**Authors:** Sara M. van Bonn, Jan S. Grajek, Tobias Schuldt, Sebastian P. Schraven, Armin Schneider, Stefanie Rettschlag, Tobias Oberhoffner, Nora M. Weiss, Robert Mlynski

**Affiliations:** 1 Klinik und Poliklinik für Hals-Nasen-Ohrenheilkunde, Kopf- und Halschirurgie „Otto Körner“, Universitätsmedizin Rostock, Doberaner Str. 137–139, 18057 Rostock, Deutschland; 2 Fachbereich Ingenieurwissenschaften, Jade Hochschule, Friedrich-Paffrath-Straße 101, 26389 Wilhelmshaven, Deutschland; 3 Klinik für Hals-Nasen-Ohrenheilkunde, Kopf‑ und Halschirurgie, Head and Neck Surgery, St. Elisabeth Hospital, Ruhr University Bochum, Bleichstr. 15, 44787, Bochum, Deutschland

**Keywords:** 3‑D-Visualisierung, Medizinische Ausbildung, Hals-Nasen-Ohren-Heilkunde, Kommunikation, Live-Operation, 3D visualization, Medical education, Otorhinolaryngology, Communication, Live surgery

## Abstract

**Hintergrund:**

Durch die Entwicklung technologischer Innovationen haben sich nicht nur das gesellschaftliche Leben und das Gesundheitssystem verändert, sondern auch die Anforderungen an die Lehre. Ziel dieser Pilotstudie war es zu evaluieren, ob Studierende bei der Observation eines mikrochirurgischen Eingriffs am Schläfenbein mit Annotation chirurgischer Landmarken ein zusätzliches Verständnis anatomischer Strukturen erlangen, wenn eine Visualisierung verwendet wird, die für Chirurg und Betrachter gleiche 3‑D-Ansichten bietet.

**Material und Methoden:**

Während regulärer Anwesenheitspraktika wurden Studierende drei Gruppen randomisiert zugewiesen: Kontroll‑, 2‑D‑ oder 3‑D-Gruppe. Mithilfe von Evaluationsbögen/intraoperativer Annotation chirurgischer Landmarken des Operationssitus erfolgte die Beurteilung des subjektiv didaktischen Werts verschiedener Visualisierungsformate und daraus resultierender Lernerfahrungen.

**Ergebnisse:**

47 Studierende konnten in die Studie einbezogen werden. Die Mehrheit der Studierenden gab einen sehr hohen Mehrwert der 3‑D-Visualisierung bezüglich der Methode (70 %) und der Anschaulichkeit (80 %) im Vergleich zur 2‑D‑ und Kontrollgruppe an. 69 % der Studierenden stimmten der Aussage voll und ganz zu, dass die 2‑D- und 3‑D-Visualisierung die Lernmotivation erhöht und sehr gut geeignet ist, Topographie/und Strukturen besser zu erkennen.

**Schlussfolgerung:**

Die Verwendung interaktiver Visualisierungsmöglichkeiten in der Lehre unterstützt die Lerneffizienz und ‑motivation der Studierenden. Besonders die 3‑D-Visualisierung und die intraoperative Annotation des Operationssitus ist ein nützliches Werkzeug in der Lehre und erhöht die Qualität dieser. Sie unterstützt die Wahrnehmung der anatomischen Topographie und ermöglicht eine gezieltere chirurgische Ausbildung.

## Abstract (en)

**Background:**

The development of technological innovations has not only changed social life and the healthcare system, but also affects medical education. The aim of this pilot study was to evaluate whether students, when observing a microsurgical procedure of the temporal bone, gain an additional understanding of the anatomical structures and surgical site when visualization is used that provides identical views for the surgeon and viewer in 3D.

**Materials and methods:**

During regular attendance tutorials, students were randomly assigned to three different groups: control group, 2D group, or 3D group. Evaluation questionnaires and intraoperative student annotation of the surgical view were used to assess the subjective didactic value of different visualization formats and resultant learning experiences for the students.

**Results:**

A total of 47 students were included in the investigations during conventional attendance tutorials. The majority indicated a high added value of the 3D visualization in terms of method (70%) and vividness (80%) compared to the 2D group and self-study; 69% of the students fully agreed with the statement that 2D and 3D visualization increases learning motivation and is a very good way to improve recognition of anatomical topography and structures.

**Conclusion:**

New interactive visualization options in teaching promote learning efficiency and motivation among students. Especially 3D visualization and intraoperative annotation of the surgical view by the student is a useful didactic tool and increases the quality of clinical teaching. It supports the perception of anatomical topography and enables more focused surgical training.

Die zunehmende Digitalisierung, speziell die Entwicklung technologischer Innovationen, hat nicht nur das gesellschaftliche Leben und das Gesundheitssystem verändert, sondern auch die Anforderungen an die Gestaltung der studentischen Lehre. Die Kategorisierung von digitalen Lehr- und Lernmedien erscheint im Kontext der Vielseitigkeit und Heterogenität des Angebots sehr komplex. Zwar ist der alltägliche Umgang mit digitalen Medien in der Freizeit allgegenwärtig und wird zur Informationsfindung und Kommunikation genutzt, für eine breite Verwendung in der Bildung fehlt jedoch die individuelle Erfahrung und ein kritisch-konstruktives Verhältnis [1].

Traditionell werden Anatomie und Operationsschritte in theoretischen Vorlesungen mit bzw. ohne Ergänzung durch verschiedene Medien vermittelt. Die Vermittlung chirurgischer Fähigkeiten und Kenntnisse durch Präparationsübungen am Körperspender oder Tiermodellen erfolgt, u. a. wegen der Kosten [2], typischerweise erst während der Facharztausbildung oder noch später. Anwesenheitspraktika im Operationssaal dienen Studierenden zur Beobachtung typischer chirurgischer Eingriffe. Allerdings stellen besonders mikroskopische und feinstrukturchirurgische Eingriffe für das konzeptionelle Verständnis der 3‑D-Anatomie für die Studierenden, aber auch für die Lehrenden, eine besondere Herausforderung dar, vor allem dann, wenn Chirurg und Studierende keine identische Ansicht des Operationssitus haben [3, 4]. Insbesondere am Schläfenbein ist das Verständnis der komplexen Anatomie wichtig für Befundung bzw. Therapieauswahl [2].

Eine 3‑D-Visualisierung kann demzufolge die Ausbildung verbessern und in der Anatomie das Verständnis von räumlichen Gegebenheiten erleichtern [5–7]. Es hat sich gezeigt, dass Studierende im Anatomieunterricht, die zusätzlich zu konventionellen auch mit interaktiven 3‑D-Unterrichtsmethoden arbeiteten, bessere Ergebnisse in Prüfungen erzielten, als Studenten, die nur konventionellen Unterricht erhielten [8]. 3‑D-Modelle gewinnen in verschiedenen Disziplinen an Bedeutung: Zum einen können diese in der chirurgischen Ausbildung das anatomische Verständnis unterstützten bzw. verbessern, zum anderen kann durch den Einsatz virtueller Techniken aber auch die pandemiebedingte fehlende praktische Ausbildung überbrückt werden [2, 5, 6, 9–12]. Zahlreiche Übersichtsarbeiten zeigten, dass die Verwendung von 3‑D-Modellen in der Anatomieausbildung die Leistung der Studierenden verbessert und die kognitive Belastung der Studierenden reduziert [13]. Für die medizinische Fortbildung am Schläfenbein gibt es für Weiterbildungsassistenten virtuelle Übungsmöglichkeiten. Hierbei werden in vorgefertigten Trainingsfällen des Mittelohrs Patient und Instrumente hochauflösend modelliert und auf einem 3‑D-Bildschirm visualisiert [2, 14]. Bisher gibt es jedoch nur wenige Untersuchungen zum Einsatz von interaktiver 3‑D-Visualisierung in der Operationslehre und zum Einsatz solcher Visualisierung in der Ausbildung von Studierenden im Fachgebiet der Hals-Nasen-Ohren-Heilkunde [3, 8].

Ziel dieser Pilotstudie war es, zur ersten Evaluierung eines Lehrkonzepts festzustellen, ob die Studierenden bei Teilnahme an einem mikrochirurgischen Eingriff am Schläfenbein ein zusätzliches Verständnis der anatomischen Strukturen und des chirurgischen Situs erlangen, wenn eine Visualisierung verwendet wird, die für Chirurg und Betrachter identische 3‑D-Ansichten bietet, sowie ob eine direkte Annotation des Operationssitus durch die Studierenden möglich ist. Zudem sollte orientierend geprüft werden, ob der Lernerfolg bei Verwendung drei verschiedener didaktischer Prozesse messbar ist und die subjektive Lernmotivation im Vergleich zur herkömmlichen Lehre durch Selbststudium steigt.

## Methodik

Die vorliegenden Daten wurden von Studierenden im fünften bis zehnten Semester Humanmedizin erhoben. Während regulärer Anwesenheitspraktika wurden die Studierenden, ohne vorherige Ankündigung, zufällig drei verschiedenen Gruppen zugewiesen: Selbststudium (Kontrollgruppe), 2‑D-Gruppe oder 3‑D-Gruppe.

**Figure 1:**
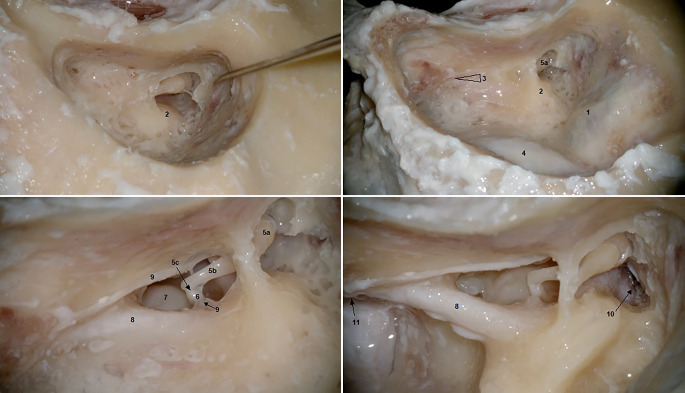

Zur Ermittlung des Wissensstands mussten alle Studierenden zehn vorgekennzeichnete anatomische Strukturen im Schläfenbein zu benennen (Tab. 1, Spalte 1; [15]). Im Anschluss wurde Informationsmaterial verteilt, bestehend aus einem Operationsbericht über eine Cochleaimplantation und dazugehöriges Bildmaterial [15, 16]. Nach Sichtung des Materials (30 min) erfolgte für die Kontrollgruppe nach Selbstlerneinheit die Wissensüberprüfung durch Benennung von elf Strukturen an ausgedruckten intraoperativen Bildern (Tab. 1, Spalte 2; Abb. 1).

**Table 1:** 

Wissensstanderhebung	Kontrollgruppe	2‑D/3-D-Gruppe
1. Mastoidzellen	1. Dura zur mittleren Schädelgrube	1. Dura zur mittleren Schädelgrube
2. Trommelfell	2. Horizontaler Bogengang	2. Horizontaler Bogengang
3. Rundes Fenster	3. Chorda-Fazialis-Winkel	3. N. Facialis (tymp. Verlauf)
4. Stapes	4. Sinus sigmoideus	4. N. Facialis (mast. Verlauf)
5. Sinus sigmoideus	5. a) Ambosskopf b) Langer Ambossfortsatz c) Processus lenticularis	5. Processus lenticularis
6. Horizontaler Bogengang	6. Stapeskopf	6. Promontorium
7. Posteriorer Bogengang	7. Promontorium	7. Stapeskopf
8. Amboss	8. N. Fazialis	8. Sinus sigmoideus
9. N. facialis	9. Chorda tympani	9. Eminentia pyramidalis
10. Hammer	10. Ganglion geniculi	10. Amboss
	11. Foramen stylomastoideum	11. Chorda tympani
		12. Ganglion geniculi
		13. Hammer
		14. Äußeres Fazialisknie

**Figure 2:**
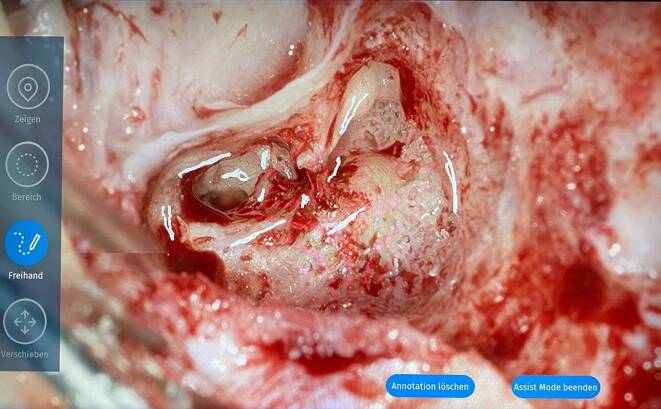

Die 2‑D- und 3‑D-Gruppen verfolgten währenddessen eine mikrochirurgische Operation im Operationssaal. Als Modelloperation wurde die Cochleaimplantation gewählt. Die Operation ist hochgradig standardisiert. Die Präparation erfolgt entlang statischer chirurgisch-anatomischer Landmarken. Die betroffenen Patient*innen haben in der Regel ein anatomisch-topographisch gesundes Schläfenbein, weshalb die Operation für die Überprüfung des Lernzuwachses bei anatomischen Bezugspunkten besonders geeignet erscheint. Innerhalb des Saals wurde die Operation mit einem volldigitalen Operationsmikroskop (Arriscope Evo2 ENT, Fa. MSI, München, Deutschland) mit der Fähigkeit, ein 2‑D- und ein 3‑D-Bild zu übertragen, demonstriert. Der Bildausschnitt, die Größe und das Operationsfeld waren für Operateur und Beobachter identisch. Die Live-Bilder wurden den Studierenden auf 65-Zoll-3-D-Displays (LG 65EF9509, Fa. LG Electronics, Seoul, Republik Korea) präsentiert. Die zwei Displays waren so positioniert, dass alle Studierenden einen direkten Blick auf die Bildschirme hatten, ohne dass es zu Blickwinkelverzerrungen kam. Alle teilnehmenden Studierenden beobachteten die chirurgischen Eingriffe entweder in 4K-Qualität in 2‑D-Ansicht (2-D-Gruppe) oder in 3‑D-Ansicht (3-D-Gruppe) mit passiven, polarisierten 3‑D-Brillen (Fa. Schleiter & Jauernig, Hamburg, Deutschland) in audiovisueller Echtzeit. Durch eine Touchscreen-Bedienoberfläche am Operationsmikroskop wurde das Betrachten und das grafische Annotieren auf dem Operationsfeld durch die Studierenden zur Markierung anatomischer Strukturen, ermöglicht („assist mode“). Alle Studierenden mussten zehn Strukturen aus dem Operationsfeld benennen (Tab. 1, Spalte 3) und diese kennzeichnen (Abb. 2). Die angezeichneten Strukturen wurden im digitalen Binokular direkt vor den Augen des Chirurgen sichtbar. Dieser konnte, ohne sich vom Binokular abwenden zu müssen, eine Aussage über richtig und falsch markierte anatomische Strukturen tätigen. Vor der Operation gaben die Patienten eine schriftliche Einverständniserklärung für die anonymisierte Übertragung der chirurgischen Bildgebung zu Lehrzwecken ab.

Nach Ende der Selbstlerneinheit sowie nach Abschluss der Live-Operation füllten die Studierenden einen Bewertungsfragebogen aus, um den subjektiven Wert der unterschiedlichen Visualisierungsmethoden zu evaluieren. Die ersten acht Fragen waren auf einer 5‑Punkte-Likert-Skala zu beantworten (volle Zustimmung = 1 Punkt; Zustimmung = 2 Punkte; unentschieden = 3 Punkte; eher keine Zustimmung = 4 Punkte; keine Zustimmung = 5 Punkte, keine Antwort möglich = 6 Punkte), und drei Aussagen waren mit einer Ratingskala und Abstufungen von sehr gut bis mangelhaft zu bewerten. Die Fragen der Evaluationsbögen sind Tab. 2 zu entnehmen. Die Ergebnisse der Auswertung wurden mit der Software Prism (Version 8, Fa. GraphPad Software, La Jolla, CA, USA) deskriptiv ausgewertet. Es wurden die Normalverteilung geprüft, Mittelwerte (M) sowie Standardabweichungen (SD) berechnet und Prozentsätze ermittelt.

**Table 2:** 

	Evaluationsbogen
1	Ich kann mit Fakten besser merken, in dem ich sie am Präparat gezeigt bekomme
2	Ich finde es hilfreich, wenn ich zu den gezeigten Bildern eine Erklärung bekomme
3	Mit der eingesetzten Lernmethode kann ich mir Fakten gut merken
4	Ich würde die eingesetzte Lernmethode gerne häufiger nutzen
5	Die eingesetzte Lernmethode motiviert mich und macht Spaß
6	Das Lernziel mit der eingesetzten Lernmethode ist klar definiert
7	Wie bewerten Sie die angewandte Lernmethode insgesamt?
8	Wie bewerten Sie die Anschaulichkeit der angewandten Lernmethode?
9	Wie bewerten Sie Ihren Lernzuwachs durch die angewandte Methode?

## Ergebnisse

**Figure 3:**
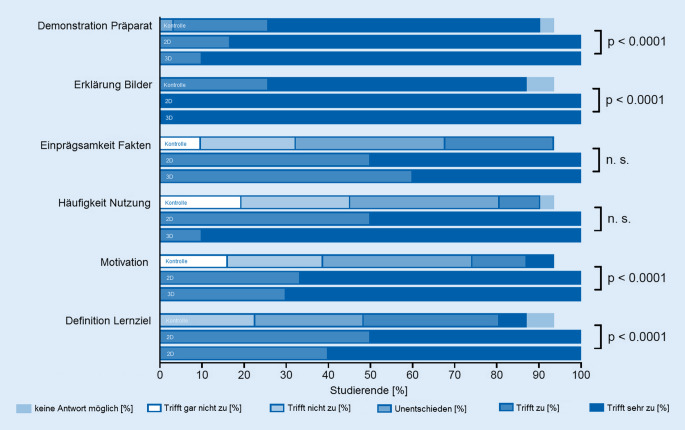

Insgesamt nahmen 47 Studierende an der Pilotstudie teil, und 45 füllten die Evaluationsbögen aus. Die jeweiligen Antworten sind Abb. 3 und 4 zu entnehmen. Alle Studierenden füllten zudem eine Wissensstanderhebung aus. Nach Aufteilung der Studierenden führten 31 das Selbststudium durch und füllten im Anschluss die Wissensüberprüfung aus. Im Operationssaal nahmen 6 Studierende in 2‑D und 10 Studierende in 3‑D an einer Cochleaimplantation teil.

**Figure 4:**
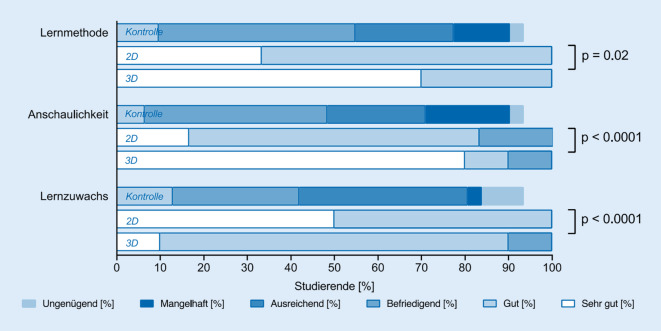

### Demonstration des Präparats

In der Kontrollgruppe stimmten der Aussage, dass Fakten einprägsamer sind, wenn sie am Präparat gezeigt werden, zwanzig Studierende (68,97 %) voll und ganz zu, sieben Studierende (24,14 %) stimmten zu und ein Studierender (3,45 %) war unentschlossen. In der 2‑D-Gruppe stimmten der Aussage fünf Studierende (83,3 %) voll und ganz zu, und ein Studierender (16,67 %) stimmte zu. In der 3‑D-Gruppe stimmten der Aussage neun Studierende (90 %) voll und ganz zu, und ein Studierender (10 %) stimmte zu.

### Erklärung der Bilder

In der Kontrollgruppe stimmten der Aussage, dass es hilfreich ist, wenn man zu gezeigten anatomischen Bildern eine Erklärung bekommt, neunzehn Studierende (65,52 %) voll und ganz zu, und acht (27,58 %) stimmten zu. In der 2‑D-Gruppe stimmten der Aussage sechs Studierende (100 %) voll und ganz zu. In der 3‑D-Gruppe stimmten der Aussage zehn Studierende (100 %) voll und ganz zu.

### Einprägsamkeit von Fakten

In der Kontrollgruppe stimmten der Aussage, dass durch die eingesetzte Lernmethode Fakten einprägsamer sind acht Studierende (27,57 %) zu, elf Studierende (37,93 %) waren unentschlossen, sieben Studierende (24,14 %) stimmten eher nicht zu, und drei Studierende (10,24 %) konnten diese Aussage nicht beurteilen. In der 2‑D-Gruppe stimmten der Aussage drei Studierende (30 %) voll und ganz zu, und drei Studierende (30 %) stimmten zu. In der 3‑D-Gruppe stimmten der Aussage vier Studierende (40 %) voll und ganz zu, und sechs Studierende (60 %) stimmten zu.

### Häufigkeit der Nutzung

In der Kontrollgruppe stimmten der Aussage, dass die eingesetzte Lernmethode häufiger genutzt werden möchte, drei Studierende (10,24 %) zu, elf Studierende (37,93 %) waren unentschlossen, acht Studierende (27,57 %) stimmten eher nicht zu, und sechs Studierende (20,69 %) konnten diese Aussage nicht beurteilen. In der 2‑D-Gruppe stimmten der Aussage drei Studierende (30 %) voll und ganz zu, und drei Studierende (30 %) stimmten zu. In der 3‑D-Gruppe stimmten der Aussage neun Studierende (90 %) voll und ganz zu, und ein Studierender (10 %) stimmte zu.

### Motivation

In der Kontrollgruppe stimmten der Aussage, dass die eingesetzte Lernmethode motivierend sei, fünf Studierende (17,24 %) voll und ganz zu, vier Studierende (13,79 %) stimmten zu, elf Studierende (37,93 %) waren unentschlossen, sieben Studierende (24,14 %) stimmten eher nicht zu, und fünf Studierende (17,24 %) konnten diese Aussage nicht beurteilen. In der 2‑D-Gruppe stimmten der Aussage 4 Studierende (66,67 %) voll und ganz zu, und zwei Studierende (33,3 %) stimmten zu. In der 3‑D-Gruppe stimmten der Aussage sieben Studierende (70 %) voll und ganz zu, und drei Studierende (30 %) stimmten zu.

### Definition des Lernziels

In der Kontrollgruppe stimmten der Aussage, dass das Lernziel mit der eingesetzten Lernmethode klar definiert sei, zwei Studierende (6,89 %) voll und ganz zu, zehn Studierende (34,48 %) stimmten zu, acht Studierende (27,58 %) waren unentschlossen, sieben Studierende (24,14 %) stimmten eher nicht zu, und zwei Studierende (6,89 %) konnten diese Aussage nicht beurteilen. In der 2‑D-Gruppe stimmten der Aussage drei Studierende (30 %) voll und ganz zu, und 3 Studierende (30 %) stimmten zu. In der 3‑D-Gruppe stimmten der Aussage sechs Studierende (60 %) voll und ganz zu, und vier Studierende (40 %) stimmten zu.

### Lernmethode

Die angewandte Methode insgesamt bewerteten in der Kontrollgruppe drei Studierende (10,34 %) Studierende mit gut, vierzehn Studierende (48,28 %) mit befriedigend, sieben Studierende (24,14 %) mit ausreichend, vier Studierende (13,79 %) mit mangelhaft und ein Studierender (3,44 %) mit ungenügend, in der 2‑D-Gruppe zwei Studierende (34 %) mit sehr gut, vier Studierende (67 %) mit gut und in der 3‑D-Gruppe sieben Studierende (70 %) mit sehr gut, drei Studierende (30 %) mit gut.

### Anschaulichkeit

Die Anschaulichkeit der angewandten Methode bewerteten in der Kontrollgruppe zwei Studierende (6,9 %) Studierende mit gut, dreizehn Studierende (44,83 %) mit befriedigend, sieben Studierende (24,14 %) mit ausreichend, sechs Studierende (13,79 %) mit mangelhaft und ein Studierender (3,44 %) mit ungenügend, in der 2‑D-Gruppe ein Studierender (17 %) mit sehr gut, vier Studierende (67 %) mit gut, ein Studierender (17 %) mit befriedigend und in der 3‑D-Gruppe acht Studierende (80 %) mit sehr gut, ein Studierender (10 %) mit gut und ein Studierender (10 %) mit befriedigend.

### Lernzuwachs

Den Lernzuwachs durch die angewandte Methode bewerteten in der Kontrollgruppe vier Studierende (13,79 %) Studierende mit gut, neun Studierende (31 %) mit befriedigend, zwölf Studierende (41,38 %) mit ausreichend, ein Studierender (3,45 %) mit mangelhaft und drei Studierende (10,34 %) mit ungenügend, in der 2‑D-Gruppe drei Studierende (50 %) mit sehr gut, drei Studierende (50 %) mit gut und in der 3‑D-Gruppe ein Studierender (10 %) mit sehr gut, acht Studierende (80 %) mit gut und ein Studierender (10 %) mit befriedigend.

Im Vergleich der drei Gruppen zeigten sich signifikante Unterschiede zwischen der Kontrollgruppe und der 2‑D- sowie 3‑D-Gruppe bei den Fragen 1 (*p* < 0,0001), 2 (*p* < 0,0001), 5 (*p* < 0,0001), 6 (*p* < 0,0001), 7 (*p* < 0,0001) und bei den Aussagen 8 (*p* = 0,02), 9 (*p* < 0,0001), und 10 (*p* < 0,0001; Abb. 3 und 4).

### Wissensstanderhebung vs. Lernerfolgskontrolle

In der Kontrollgruppe haben die Studierenden in der Wissensstanderhebung 180 (58 %) richtige und 130 (42 %) falsche Antworten gegeben. In der Lernerfolgskontrolle waren 221 (51 %) Antworten richtig und 213 (49 %) falsch. In der 2‑D-Gruppe haben die Studierenden in der Wissensstanderhebung 48 (80 %) richtige und 12 (20 %) falsche Antworten gegeben. In der Kontrolle am Arriscope wurden 36 (69 %) Strukturen richtig und 25 (31 %) falsch eingezeichnet. In der 3‑D-Gruppe haben die Studierenden in der Wissensstanderhebung 70 (70 %) richtige und 30 (30 %) falsche Antworten gegeben. In der Kontrolle am Arriscope wurden 47 (47 %) Strukturen richtig und 53 (53 %) falsch eingezeichnet.

## Diskussion

Durch eine zunehmende Digitalisierung hat sich nicht nur das gesellschaftliche Leben, sondern auch die Anforderungen an eine moderne Gesundheitsversorgung und Revolutionierung der Lehre verändert. Innerhalb der letzten Jahre haben neue technologische Innovationen zunehmend die Medizin und Lehre erreicht und somit ebenso die Anforderungen an der Durchführung neuartiger Lehrmethoden verändert [17]. Die konventionelle Wissensaneignung mit Lehrbuch und bibliothekarischem Lernen scheint veraltet. Moderne Lehrformate haben den Anspruch, traditionellen Lehrmethoden überlegen zu sein. Beispielsweise wird durch die Digitalisierung in der Lehre nachweislich die Lernmotivation gesteigert [18, 19]. Bei der Erprobung neuer Lehrmethoden ist neben objektiven Verfahren auch der subjektive Nutzen von großer Bedeutung, da besonders die individuelle Wahrnehmung des Lernens durch die Studierenden als ein Hauptindikator für den Lernerfolg angesehen wird [3, 20, 21]. Das Fachgebiet der Hals-Nasen-Ohren-Heilkunde ist breit gefächert und aufgrund hervorragender Visualisierungsmöglichkeiten besonders gut für die Entwicklung neuer Bildungsressourcen geeignet [22, 23]. Studierende zeigen insgesamt ein hohes Interesse an der Modernisierung und Digitalisierung in der HNO-Ausbildung [23].

Für das konzeptionelle Verständnis der Anatomie ist das Erlernen von 3‑D-Beziehungen im Operationsfeld, der Orientierung zwischen Mikrostrukturen und individuellen chirurgischen Proportionen von großer Bedeutung [24, 25]. Studien haben gezeigt, dass digitale 3‑D-Modelle eine wertvolle Ergänzung zu bestehenden Lehrmethoden, insbesondere für komplizierte mikrochirurgische Strukturen, sein können [7, 26, 27]. Die Mikrochirurgie des Schläfenbeins setzt ein präzises Arbeiten sowie ein detailliertes anatomisches Wissen zwingend voraus.

Die meisten mikrochirurgischen Eingriffe werden mit konventionellen optischen Operationsmikroskopen durchgeführt, die eine stereoskopische Sicht mit binokularem Sehen für den Operateur durch das Mikroskop ermöglichen. Die Übertragung der Operation für die Studierenden erfolgt durch einen zusätzlichen Strahlenteiler, entweder monokular oder an eine Kamera gekoppelt, um auf einem separaten Bildschirm die Operation binokular verfolgen zu können. Das binokulare Sehen ist dem monokularen überlegen. Studien zeigen, dass bei Probanden, denen zudem die Möglichkeit der Stereovision fehlt, das binokulare nichtstereoskopische Sehen beim Erlernen chirurgischer Fertigkeiten von Vorteil ist [28]. Für die Studierenden ist es einfacher, die Operationsschritte und Kommentare des Operateurs nachvollziehen zu können, wenn sie die gleiche Sicht wie der Chirurg auf das Operationsgebiet haben. Die Verbesserung der chirurgischen Visualisierung stellt einen hohen Mehrwert für die Qualität der Lehre dar [3, 7].

80 % der Studierenden bewerteten die Überlegenheit der 3‑D-Visualisierung gegenüber der 2‑D-Visualisierung hinsichtlich der Wahrnehmung anatomischer Topographie und Strukturen und 70 % der Studierenden die 3‑D-Lernmethode mit sehr gut. Auch gaben die Studierenden eine subjektiv gesteigerte Einprägsamkeit gezeigter Strukturen und Lernmotivation an. Für einen messbaren Vorteil im Lernzuwachs für die Benennung anatomischer Landmarken trifft dies allerdings nicht zu. Hinsichtlich der Wissensstanderhebung und Lernkontrolle/Benennung chirurgisch-anatomischer Landmarken im Assistentenmodus zeigten sich unterschiedliche, teils stark variierende und nicht vergleichbare Ergebnisse. Dies ist darauf zurückzuführen, dass die Studierenden verschiedenen Semestern zugehörig waren, sie einen unterschiedlichen Kenntnisstand hatten und die Stichprobengrößen für die Komplexität der Anatomie möglicherweise zu klein sind. Zur Messung des Lernzuwachses in Abhängigkeit von der Methode sind die Testgruppen bezüglich ihres Kenntnisstands vor der Testung bei zukünftigen Studien zu nivellieren. Die vorliegenden Untersuchungen zeigen, dass der Lernzuwachs bei den Studierenden unterschiedlich und stark von verschiedenen Faktoren (Vorkenntnisse, Eigenmotivation) abhängig ist. Praktische Fertigkeiten für angehende Otochirurgen werden nicht vermittelt. Psychomotorische Lernstrukturen sind abhängig von der Anzahl der praktizierten Repetitionen, wie Untersuchungen zu Fertigkeiten bei der HNO-Spiegeluntersuchung zeigen [29]. Als „Outcome-Parameter“ zur Evaluation einer solchen Lehrmethode kann der Lernzuwachs für praktische Fertigkeiten nur bedingt herangezogen werden.

Die Evaluation zeigte richtungsweisend, dass die verwendeten Visualisierungsmöglichkeiten das Interesse der Studierenden steigerten und somit zu einem subjektiv bedeutenden Nutzen und langfristig zu einer Motivationssteigerung führt [18, 20, 30]. Zudem zeigen die Ergebnisse, dass der gefühlte Lernzuwachs für die Studierenden wesentlich höher ist, wenn die gezeigte Operation in persönlicher Interaktion mit dem Operateur und Erklärungen am Bildschirm in hoher Bilddarstellungsqualität erfolgt. Im Vergleich zum Selbststudium zeigten sich die Studierenden der 2‑D- und 3‑D-Gruppe deutlich zufriedener und damit auch motivierter. Der Zuwachs an didaktischer Qualität ist signifikant höher bei dieser Form der Lehre. Zwischen den beiden interaktiven Gruppen 2‑D/3-D lässt sich mit der verwendeten Evaluation kein signifikanter Unterschied in den Ergebnissen der subjektiven Qualität der Lehrmethode nachweisen. Die Vorteile der Verwendung hochauflösender Echtzeit-Bilddarstellung gegenüber papierbasierter Lehre von Operationsverfahren ist möglicherweise derartig groß, dass sie viel leichter messbar sind gegenüber den didaktischen Unterschieden der 2‑D- und 3‑D-Darstellung. Für den Nachweis des didaktischen Vorteils von 3‑D- gegenüber 2‑D-basierter visueller Lehre sind speziellere Testdesigns für die verblindete Testung und/oder der direkte Vergleich der Visualisierung für die Testpersonen wahrscheinlich geeigneter. Eine weitere Limitation dieser Pilotstudie ist die geringe Anzahl an teilnehmenden Studierenden, besonders bezüglich der 2‑D- und 3‑D-Gruppen, die in die Untersuchung eingeschlossen werden konnten. Dies ist bedingt durch die Einschränkungen des Präsenzunterrichts und die Reduktion des elektiven Operationsprogramms aufgrund der COVID-19-Pandemie. Um diesen Limitationen zu entgegnen, soll in einem nächsten Schritt die Etablierung des multimodalen Lehrkonzepts der 3‑D-Visualisierung anhand einer größeren Gruppe von Studierenden eines gesamten Semesters verglichen werden.

Vor dem Hintergrund der durch die COVID-19-Pandemie resultierenden Einschränkungen des Präsenzunterrichts zeigen die vorliegenden Ergebnisse, dass die Studierenden auch mit privaten Endgeräten von zu Hause aus in 2‑D-Ansicht an einer Operation teilnehmen könnten. Neben innovativen Lehrformaten, erzwungen durch Kontaktbeschränkungen, lässt sich schlussfolgern, den technischen Vorteil für zentrale Lehrformate zu nutzen [31–34]. Strukturschwache Länder könnten von der zentralen Ausstrahlung audiovisueller Inhalte mit direkter Interaktion für die Studierenden besonders profitieren [35]. Beispielsweise können seltene oder zentralisierte Operationsverfahren sowie Behandlungen aus Schwerpunktkliniken für die Ausbildung transparent gemacht werden. Da sich sowohl für die 2‑D- als auch die 3‑D-Visualisierung ein positiver Effekt zeigte, gilt es sicherzustellen, dass diese visuellen interaktiven Technologien integraler und zugänglicher Bestandteil regulärer Lehrmethoden werden. Die technische Ausstattung der ausbildenden Klinika und Universitäten ist entsprechend anzupassen. Sie stellt eine effiziente Methode dar, die das umfangreiche Selbststudium unterstützt [19, 36].

## Fazit für die Praxis

- Interaktive digitale Modelle und unterschiedliche Visualisierungsmöglichkeiten verbessern und unterstützen bestehende Lehrmethoden.
- Die Resultate zeigen eine große Akzeptanz bei den Studierenden.
- Durch neuartige Visualisierungsmöglichkeiten wird das Interesse der Studierenden gesteigert und führt zu einem subjektiv starken Nutzen und somit langfristig auch zu einer Motivationssteigerung.
- Visuelle interaktive Technologien sollten ein integraler Bestandteil des regulären Lehrplans sein.
